# Speciation Success of Polyploid Plants Closely Relates to the Regulation of Meiotic Recombination

**DOI:** 10.3389/fpls.2018.00907

**Published:** 2018-06-28

**Authors:** Alexandre Pelé, Mathieu Rousseau-Gueutin, Anne-Marie Chèvre

**Affiliations:** ^1^Plant Breeding, Wageningen University & Research, Wageningen, Netherlands; ^2^Institut de Génétique, Environnement et Protection des Plantes, Institut National de la Recherche Agronomique, Agrocampus Ouest, Université de Rennes 1, Rennes, France

**Keywords:** polyploidy, genome evolution, diploidization, meiosis, unreduced gametes, recombination, crossover

## Abstract

Polyploidization is a widespread phenomenon, especially in flowering plants that have all undergone at least one event of whole genome duplication during their evolutionary history. Consequently, a large range of plants, including many of the world’s crops, combines more than two sets of chromosomes originating from the same (autopolyploids) or related species (allopolyploids). Depending on the polyploid formation pathway, different patterns of recombination will be promoted, conditioning the level of heterozygosity. A polyploid population harboring a high level of heterozygosity will produce more genetically diverse progenies. Some of these individuals may show a better adaptability to different ecological niches, increasing their chance for successful establishment through natural selection. Another condition for young polyploids to survive corresponds to the formation of well-balanced gametes, assuring a sufficient level of fertility. In this review, we discuss the consequences of polyploid formation pathways, meiotic behavior and recombination regulation on the speciation success and maintenance of polyploid species.

## Introduction

Meiosis is the fundamental process by which are formed the gametes in all sexual organisms. Largely investigated in the last decades (for review see Mercier et al., 2015; Zickler and Kleckner, 2015), this process consists in a single phase of DNA replication followed by two divisions, where first, pairs of parental chromosomes (i.e., homologs) and then, sister chromatids separate into four cells of a tetrad. During the first division, occurrence of meiotic recombination is determinant for ensuring both genome stability and generation of diversity, through one of its products: the crossovers. Indeed, in addition to maintain pairs of homologs physically linked at the end of metaphase I (i.e., bivalents), crossovers result in reciprocal exchanges of DNA between non-sister chromatids. At least one crossover is required per bivalent to obtain well-balanced gametes and avoid the formation of aneuploid progenies. However, as a result of the so-called phenomenon of interference, rarely more than three crossovers are formed per bivalent in a meiosis, typically widely spaced from one another and primarily located on chromosome extremities.

In polyploids, which are widespread in plants even in major crops (e.g., cotton, rapeseed, and wheat), the situation is delicate as they combine two genomes or more deriving from the same (autopolyploidy) or related (allopolyploidy) species (Stebbins, 1947). While all Angiosperms have experienced at least one event of whole genome duplication in their evolutionary history (Jiao et al., 2011), polyploidization remains an active and ongoing process recognized as a major driving force for plants speciation (Comai, 2005; Alix et al., 2017). Indeed, polyploids may occupy new ecological niches (Stebbins, 1985; Blaine Marchant et al., 2016) and often display higher adaptability than their progenitors, as evidenced by their better tolerance to abiotic stresses (McIntyre, 2012; Allario et al., 2013). However, the reasons of such a speciation success are not well-understood given that polyploidization initially results in a depletion of variability, due to the limited number of parental genotypes, and frequently confers instant reproductive isolation (Husband and Schemske, 2000; Husband and Sabara, 2004). In this review, we aim to highlight how meiotic recombination may favor this success by (1) conditioning the genetic variability of newly formed polyploids, (2) expanding the allelic combinatorial possibilities in the following generations, and (3) ensuring the proper segregation of multiple homologs and/or related chromosomes (i.e., homoeologs) in established auto- and allopolyploids, respectively.

## The Routes Leading to Polyploidy Combined With the Occurrence of Meiotic Recombination Condition the Initial Allelic Variability

**FIGURE 1:**
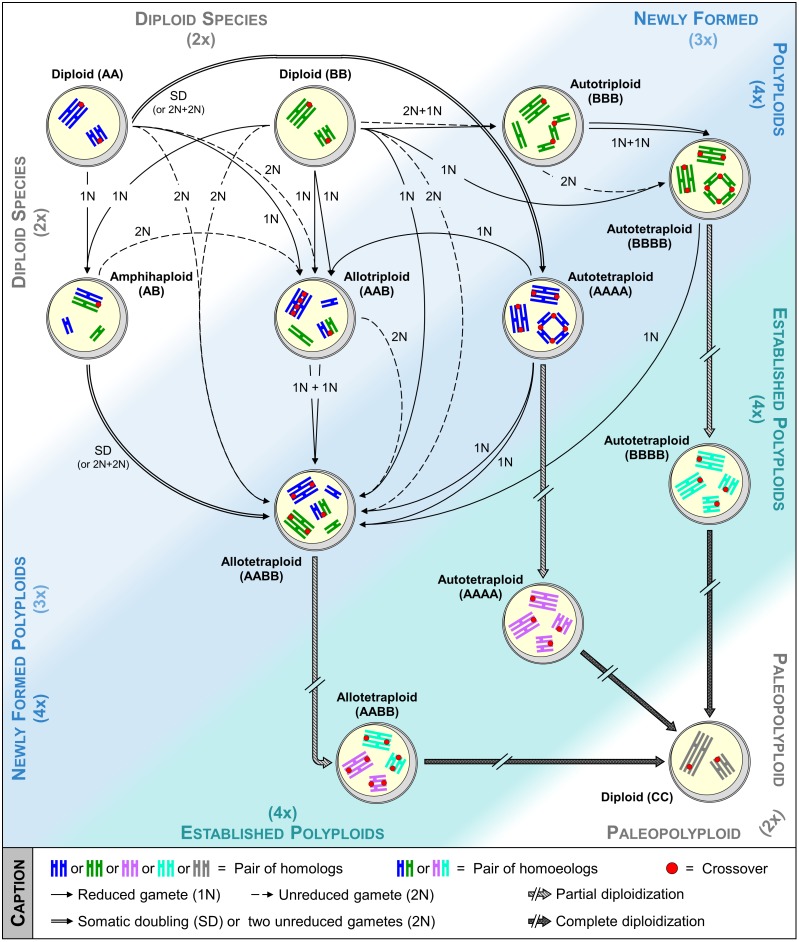
From the formation toward the complete diploidization of polyploids: insights of meiotic pairing and recombination. Auto- and allotetraploids can be formed *via* many routes, which may involve intermediates (e.g., triploids and amphihaploids). While pairing and recombination are initially perturbed, as represented by multiple/illegitimate associations and by increasing number of crossovers, a diploid-like meiosis is setting up through a strict regulation of meiotic recombination, a process referred as partial diploidization. Following millions of years, diploidization becomes complete through a return at a diploid stage resulting in a chromosome number reduction. On this figure, insights of pairing and recombination mostly based on research performed in *Arabidopsis*, *Brassica*, *Lolium*, and *Phlox* genera are presented. Within each pollen mother cell drawn, the chromosomes of the same size and same color derive from the same species, while those of different colors derive from two different species.

A novel polyploid individual may form *via* several routes (Ramsey and Schemske, 1998; Tayalé and Parisod, 2013) (**Figure 1**). Depending on the formation pathway, the genetic status (i.e., level of heterozygosity vs. homozygosity) of newly formed polyploids will highly differ and may impact their performance and speciation success, as evidenced by gains recorded in highly heterozygous plants for growth, fertility, and yield (Bingham, 1980; Stebbins, 1980; Werner and Peloquin, 1991). While immediate consequences of polyploidization were mostly investigated by inducing the somatic doubling of chromosomes through chemical treatment (Tamayo-Ordóñez et al., 2016), this path does not fully mimic what happened in nature in terms of occurrence and variability. Indeed, although possible when mitotic non-disjunction of sister-chromatids arises either in meristem tissue of sporophytes, zygote or young embryo, this route remains rarely observed spontaneously and restricts the number of alleles fixed per locus in auto- and allotetraploids (Ramsey and Schemske, 1998).

**FIGURE 2:**
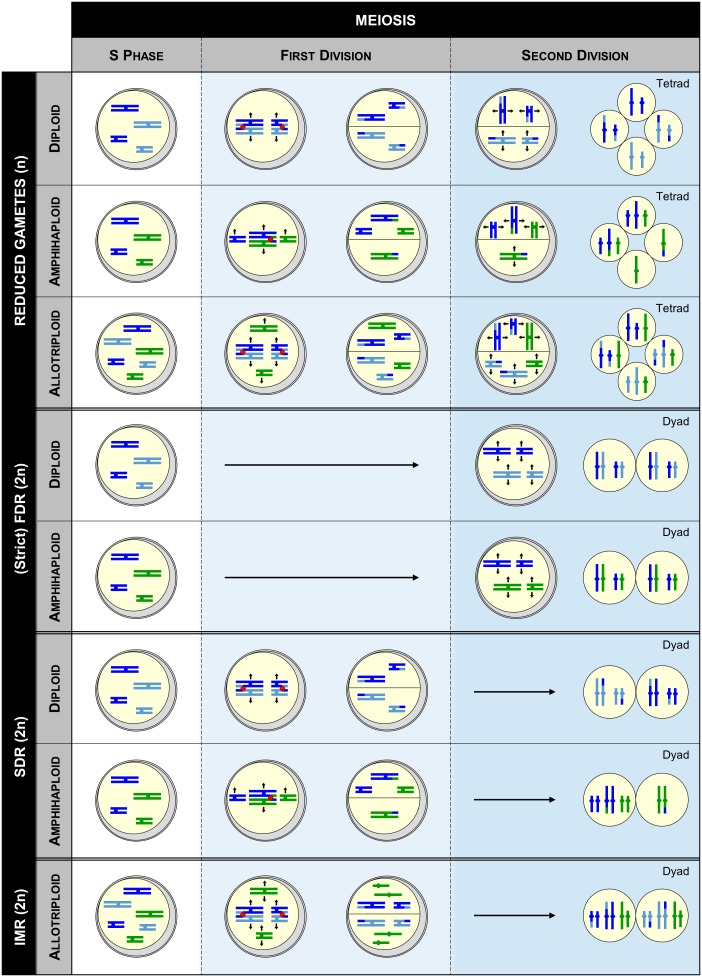
Genotypic outcome of gametes deriving from diploid, amphihaploid and allotriploid heterozygous individuals when meiosis either occurs normally (WT) or undergoes a meiotic restitution (FDR, SDR, or IMR). FDR, SDR, and IMR correspond to first, second, and intermediate meiotic restitution, respectively. Within each pollen mother cell drawn, the chromosomes of the same size colored in light and dark blue derive from the same species (differentiation showing the heterozygosity), while those colored in blue and green derive from two different species. Note that this figure is not exhaustive, as other mechanisms such as FDR-like may occur (see the text).

Nowadays, it is accepted that polyploids predominantly arise sexually, through the generation of gametes having the somatic (2n) rather than the haploid (n) number of chromosomes; a phenomenon referred as ‘gametic non-reduction’ (Harlan and de Wet, 1975). Indeed, production of unreduced gametes has been observed across widely disparate phyla (Veilleux, 1983; Bretagnolle and Thompson, 1995), at frequencies typically averaging from 0.1 to 2.0% in natural populations (Kreiner et al., 2017). Moreover, polyploidy induction may have been facilitated throughout plants evolutionary history *via* greatly enhanced frequencies of unreduced gametes. For instance, abiotic stresses such as temperature fluctuation often favor the production of unreduced gametes (Mason et al., 2011; Pécrix et al., 2011; De Storme et al., 2012), which is striking in regard to the coincidence of ancient WGD events with adverse climatic events (Vanneste et al., 2014; Van de Peer et al., 2017). On the other hand, mutation of certain genes may also have facilitated polyploidization, especially when promoting unreduced gametes in both male and female meiosis, as observed for *OSD1* and *TAM* in *Arabidopsis thaliana* (d’Erfurth et al., 2009, 2010; Wang et al., 2010). Although a plethora of cytologic mechanisms has been described (De Storme and Geelen, 2013), unreduced gametes commonly arise in plants through First (FDR) or Second Division Restitution (SDR), corresponding to the defect of meiosis I or II, respectively. Thus, depending on their origin, unreduced gametes will display different genetic makeups (Bretagnolle and Thompson, 1995; Brownfield and Köhler, 2011). In the strict sense, the non-disjunction of homologs in FDR is combined with the abolishment of recombination, yielding unreduced gametes retaining the full heterozygosity of the initial individual (**Figure 2**); note however that in some instance a partial loss of variability happens due to the occurrence of recombination, a mechanism referred as FDR-like (Ramanna and Jacobsen, 2003). In contrast, SDR, consisting in the exclusive separation of recombined homologs, always results in partially homozygous unreduced gametes, from the crossover location toward the end of a chromosome arm (**Figure 2**).

Two major routes may lead to the formation of both auto- and allotetraploids, either directly or indirectly *via* a triploid-bridge (**Figure 1**). Theoretically, the highest level of variability is obtained when tetraploids arise directly from the merger of two unreduced gametes provided by different diploid individuals, especially when they belong to the (strict) FDR-type. Indeed, increasing proportions of tri- and tetra-alleles per locus are expected in autotetraploids, while allotetraploids systematically benefit from a full heterozygosity between homologs and homoeologs (Watanabe et al., 1991). Nevertheless, a partial loss of variability may occur between homologs when tetraploids originate from SDR (or FDR-like) unreduced gametes. Except in case of spontaneous mutation preventing the separation of homologs or chromatids in male and female meiosis, two unreduced gametes have relatively low chance to merge in a single step (Ramsey and Schemske, 1998). Therefore, even though a lower variability is expected by this route, it is suggested that tetraploid species are most presumably formed in two steps *via* a triploid bridge (Husband, 2004). Indeed, triploids resulting through the merger of reduced and unreduced gametes are fertile in a large range of plant lineages (Ramanna and Jacobsen, 2003) and may form a tetraploid either by self-fertilization or intercross with one diploid progenitor. From the first path (self-fertilization), two reduced gametes of the triploid individual are required. However, the resulting plants are often aneuploids, decreasing the likelihood of successful speciation (**Figure 2**; Leflon et al., 2006). From the second path (intercross), the triploid must provide an unreduced gamete, likely arising from an indeterminate meiotic restitution (IMR), where FDR and SDR occur simultaneously for unpaired and paired chromosomes, respectively (**Figure 2**; De Storme and Geelen, 2013).

Allotetraploids may also arise from amphihaploid or autotetraploid intermediates, most presumably in a single step, even though both can first promote an allotriploid bridge (**Figure 1**). However, these paths may have a low natural occurrence. Indeed, while amphihaploids are generally poorly fertile (Mason and Pires, 2015), the success of allotetraploid formation through an autotetraploid closely relates to the ability of this latter to generate well-balanced reduced gametes (see the following sections). The path by which the autotetraploids arose conditions the variability per subgenome of the resulting allotetraploid. On the other hand, the amphihaploid path will provide a lower variability than all routes previously described as only one chromatid of each chromosome is assigned per dyad through (strict) FDR-type, maintaining exclusively the heterozygosity between homoeologs (**Figure 2**). Even more harmful, SDR-type (and FDR-like) may result in unbalanced dyads due to the occurrence of crossovers between homoeologs (Cifuentes et al., 2010).

## Managing the Variability in Newly Formed Polyploids Through Disturbed Meiotic Recombination

Although newly formed polyploids may sometimes successfully establish *via* the main use of vegetative reproduction, as in the young allodecaploid *Spartina anglica* (Ainouche et al., 2004), most favor sexual reproduction (Comai, 2005; Nasrallah, 2017). In addition to harboring a higher variability (**Figure 1**), populations deriving from this latter route will benefit from different patterns of meiotic recombination, increasing their chance of adaptation in new ecological niches and their speciation success through natural selection.

While the generation of diversity is usually hampered, polyploid plants are often able to overcome the limits arising from the tight regulation of meiotic recombination (Mercier et al., 2015). Indeed, as reported in *Arabidopsis*, *Brassica*, *Gossypium*, *Phlox*, and *Zea* genera, newly formed auto- and allotetraploids exhibit higher crossover frequencies between their homologous chromosomes than their diploid progenitors (Bingham et al., 1968; Raghuvanshi and Pathak, 1975; Desai et al., 2006; Leflon et al., 2010; Pecinka et al., 2011). For instance, in allotetraploids *Brassica napus* (AACC, 2*n* = 4x = 38), resulting from the hybridization of *Brassica rapa* (AA, 2*n* = 2x = 20) and *Brassica oleracea* (CC, 2*n* = 2x = 18) (Nagaharu, 1935), about twice as many crossovers were detected between A homologs than in diploid AA plants; whilst both displayed identical A genotypes (Leflon et al., 2010). Similarly, substantial increase of crossover frequencies was found in resynthesized triploids, typically lower than in tetraploids (Gymer and Whittington, 1975; Raghuvanshi and Pathak, 1975), but astonishingly more elevated in *Brassica* AAC allotriploids (Leflon et al., 2010). Moreover, Pelé et al. (2017) have recently pointed out that this crossovers boost occurring within the A genome was strikingly associated with reduced interference and dramatic changes in the shape of recombination landscapes. While the molecular mechanisms remain unknown, observations made in *Brassica* aneuploids suggest that this phenomenon is genetically controlled. Indeed, Suay et al. (2014) demonstrated that the boost of crossovers arising in AAC allotriploids relates to specific additional C chromosomes; the single C09 explaining 50% of the overall variation. Although exceptions have been reported in *Clitoria ternatea* and *Secale cereale* (Hazarika and Rees, 1967; Gandhi and Patil, 1997), enhanced recombination frequencies may have huge repercussions on the speciation success. The wider diversity of resulting gametes may indeed accelerate the elimination of deleterious alleles and facilitate in the long run adaptation of neopolyploids to adverse environmental situations.

In newly formed allopolyploids, meiotic recombination may also occur between the homoeologous chromosomes, as reported in diverse species including *Brassica napus*, *Coffea arabica*, *Nicotiana tabacum*, and *Tragopogon miscellus* (Song et al., 1995; Lim et al., 2004; Gaeta et al., 2007; Gaeta and Chris Pires, 2010; Xiong et al., 2011; Chester et al., 2012; Lashermes et al., 2014). Detected as early as the first meiosis of resynthesized allotetraploids (Szadkowski et al., 2010), homoeologous recombination frequency often correlates with the existing collinearity between homoeologs and varies according to the route of polyploid formation (Szadkowski et al., 2011; Rousseau-Gueutin et al., 2016). For instance, while such events are almost inexistent in the previously described *Brassica* AAC allotriploids (Leflon et al., 2006; Pelé et al., 2017), they commonly occur in ACC allotriploids and AC amphihaploids (Cifuentes et al., 2010; Yang et al., 2017). Moreover, the resulting homoeologous exchanges are smaller and more frequent when arising from unreduced gametes of amphihaploids rather than by somatic doubling (Szadkowski et al., 2011). These homoeologous exchanges deeply impact the variability and gene content of newly formed polyploids. For instance, the young allotetraploid *Coffea arabica* showed about 5% of homoeologous gene loss since its formation (Lashermes et al., 2014). Even more astonishing, up to 10% of genes are impacted after only three generations following the resynthesis of *Brassica napus* (Rousseau-Gueutin et al., 2016), highlighting that homoeologous exchanges are a major cause of gene copy number variation in *Brassica napus* varieties (Hurgobin et al., 2018). In some instances, these structural changes are at the origin of phenotypic variations, such as flowering time divergence, seed quality or disease resistance (Pires et al., 2004; Zhao et al., 2006; Stein et al., 2017), which may have contributed in the ability of allopolyploid species to exploit a wider range of environmental conditions.

## Ensuring a Diploid-Like Meiosis to Get Fully Established Through Overall or Targeted Depletion of Meiotic Crossovers

The presence of more than one possible partner to pair and recombine with may however lead to the generation of unbalanced gametes and reduced fertility, whenever illegitimate or multiple associations arise between chromosomes at Metaphase I of meiosis (Ramsey and Schemske, 2002). Nevertheless, while commonly (but not systematically) observed in resynthesized polyploids, such associations unfrequently occur in the established ones (**Figure 1** and Supplementary Table 1). Considering the contrasted examples summarized in Supplementary Table 1, it seems that the mechanisms leading to a diploid-like meiosis (referred as ‘partial diploidization’) may either already exist in the parental diploids or are set up after the polyploid formation. Indeed, some species show a global genome stasis since their first meiosis (see *Gossypium hirsutum*), while others revealed increasing proportion of bivalents in the following generations (see *Arabidopsis thaliana*, *Pennisetum typhoides*). Although, this may be species-specific, the partial diploidization requires a particular regulation of meiotic recombination that differs according to the polyploids type.

In autopolyploids, multiple copies of every chromosome are true homologs thereby sharing the same chance to pair and recombine with each other. Consequently, when all homologs align in parallel during the Prophase I of meiosis, multiple associations may occur (Lloyd and Bomblies, 2016). However, while these associations are dissolved prior to Metaphase I in established autopolyploids, which primarily form bivalents through a random assortment of homologs into pair (i.e., polysomic inheritance), they are frequently maintained in those resynthesized that exhibit trivalents and/or tetravalents (Supplementary Table 1). Theoretically, a sharp reduction in the overall number of crossovers can overcome this fate, especially by ensuring a single crossover per chromosome (Lloyd and Bomblies, 2016). Although exceptions were reported, this theory has gained concrete support in the autotetraploid *Arabidopsis arenosa*. Indeed, while multivalents and increased crossover rates are observed following polyploidy induction, natural accessions exhibit predominantly bivalents with on average 1.09 crossover (Carvalho et al., 2010; Pecinka et al., 2011; Yant et al., 2013). The molecular basis of the overall crossover number reduction in established autopolyploids remains unknown but it is suggested to result from elevated interference given that the obligatory crossover is maintained per homolog pair (Bomblies et al., 2016). Additionally, genomic comparison of *Arabidopsis arenosa* and its related diploids evidenced the selection of a few meiotic genes involved in the process of crossover formation, thereby providing a list of candidates to test (Yant et al., 2013).

In allopolyploids the situation is even more challenging because of their hybrid origin. Indeed, generation of balanced gametes requires that chromosomes form pairs, instead of multivalents, and that pairs are restricted to homologs (i.e., disomic inheritance). Targeted rather than overall reduction in the number of crossovers is therefore more relevant for dissolving illegitimate associations occurring when homoeologs align in parallel during the Prophase I (Lloyd and Bomblies, 2016). Consistently, allopolyploids seem to maintain elevated crossover rates between their homologs throughout their evolution. Indeed, like resynthesized allotetraploids, cultivars of *Brassica napus* show twice more crossovers than related diploids (Wang et al., 2012; Chalhoub et al., 2014; Cai et al., 2017). Although efficient, homoeologs recognition is not completely error proof as small homoeologous exchanges may be detected in modern allopolyploids (Lloyd et al., 2018), but to a lesser extent than in the resynthesized allopolyploids (Supplementary Table 1). Previously thought to result from the increased divergence between homoeologous genomes (Feldman et al., 1997), it is now considered that this process is more likely genetically controlled (Jenczewski and Alix, 2004). So far, only the *Pairing homoeologous 1* (*Ph1*) locus acting in the hexaploid bread wheat (*Triticum aestivum*, AABBDD, 2*n* = 6x = 42) has been molecularly characterized (Sears, 1976; Griffiths et al., 2006). Briefly, this latter corresponds to a cluster of defective *cyclin dependent kinases*-like (*CDKs*) and methyl-transferase genes, where is inserted a paralog of the major crossover gene *ZIP4* that is responsible for the *Ph1* phenotype (Knight et al., 2010; Greer et al., 2012; Martín et al., 2014, 2017). Indeed, this latter *ZIP4* copy was recently shown to promote homologous recombination while inhibiting the maturation of crossovers between homoeologs (Rey et al., 2017). Moreover, although the underlying gene remains unknown, a *Ph2* locus acting on the synapsis progression has been identified in wheat, likely promoting the *Ph1* efficiency rather than directly suppressing homoeologs crossovers (Martinez et al., 2001; Sutton et al., 2003). Finally, two further genomic regions limiting homoeologous recombination have been mapped in *Arabidopsis suecica* (*BYS*) and *Brassica napus* (*PrBn*) (Liu et al., 2006; Henry et al., 2014). However, while *BYS* explains less than 10% of the variability, the efficiency of *PrBn* in the allotetraploid *Brassica napus* remains unclear as it was detected through a segregating population of amphihaploids and may thereby act exclusively in a single dose (Nicolas et al., 2009; Grandont et al., 2014).

## Conclusion and Perspective

In this review, we showed that a particular regulation of meiotic recombination may have huge repercussions on the level of genetic diversity and genome stability of polyploids, and thereafter on their speciation success through natural selection. While the molecular basis of meiotic recombination has been strongly investigated in diploid species (for review see Mercier et al., 2015; Zickler and Kleckner, 2015), with recent discoveries of genes and factors (i.e., genomic and epigenetic) controlling formation and frequency of crossovers (Fernandes et al., 2017; Ziolkowski et al., 2017; Serra et al., 2018; Underwood et al., 2018), far less is known in polyploids. However, it has been shown that following polyploidization, duplicated copies of genes regulating meiosis and recombination process are preferentially lost (De Smet et al., 2013; Lloyd et al., 2014). Therefore, with a special attention on meiotic dosage-sensitive genes, and by taking advantage of the increasing number of sequenced polyploid plant genomes as well as of the major advances in NGS and genome editing (Crispr-Cas9) technologies, it will be possible to better understand the molecular mechanisms governing regulation of meiotic recombination in polyploids, from their formation toward their establishment. This increased knowledge on meiotic recombination will thereafter facilitate the growth of genetic diversity or introgression of gene of interest in polyploid crops.

## Author Contributions

AP organized and prepared the major part of the manuscript. A-MC and MR-G contributed to writing and reviewing the manuscript.

## Conflict of Interest Statement

The authors declare that the research was conducted in the absence of any commercial or financial relationships that could be construed as a potential conflict of interest.
